# Medicinal properties of ‘true’ cinnamon (*Cinnamomum zeylanicum*): a systematic review

**DOI:** 10.1186/1472-6882-13-275

**Published:** 2013-10-22

**Authors:** Priyanga Ranasinghe, Shehani Pigera, GA Sirimal Premakumara, Priyadarshani Galappaththy, Godwin R Constantine, Prasad Katulanda

**Affiliations:** 1 Department of Pharmacology, Faculty of Medicine, University of Colombo, Colombo, Sri Lanka; 2 Industrial Technology Institute, Colombo, Sri Lanka; 3 Department of Clinical Medicine, Faculty of Medicine, University of Colombo, Colombo, Sri Lanka

**Keywords:** *Cinnamomum zeylanicum*, True cinnamon, Ceylon cinnamon, Medicinal properties, Health benefits

## Abstract

**Background:**

In traditional medicine Cinnamon is considered a remedy for respiratory, digestive and gynaecological ailments. *In-vitro* and *in-vivo* studies from different parts of the world have demonstrated numerous beneficial medicinal effects of *Cinnamomum zeylanicum* (CZ). This paper aims to systematically review the scientific literature and provide a comprehensive summary on the potential medicinal benefits of CZ.

**Methods:**

A comprehensive systematic review was conducted in the following databases; PubMed, Web of Science, SciVerse Scopus for studies published before 31st December 2012. The following keywords were used: “*Cinnamomum zeylanicum*”, “Ceylon cinnamon”, “True cinnamon” and “Sri Lankan cinnamon”. To obtain additional data a manual search was performed using the reference lists of included articles.

**Results:**

The literature search identified the following number of articles in the respective databases; PubMed=54, Web of Science=76 and SciVerse Scopus=591. Thirteen additional articles were identified by searching reference lists. After removing duplicates the total number of articles included in the present review is 70. The beneficial health effects of CZ identified were; a) anti-microbial and anti-parasitic activity, b) lowering of blood glucose, blood pressure and serum cholesterol, c) anti-oxidant and free-radical scavenging properties, d) inhibition of tau aggregation and filament formation (hallmarks of Alzheimer’s disease), e) inhibitory effects on osteoclastogenesis, f) anti-secretagogue and anti-gastric ulcer effects, g) anti-nociceptive and anti-inflammatory activity, h) wound healing properties and i) hepato-protective effects. The studies reported minimal toxic and adverse effects.

**Conclusions:**

The available *in-vitro* and *in-vivo* evidence suggests that CZ has many beneficial health effects. However, since data on humans are sparse, randomized controlled trials in humans will be necessary to determine whether these effects have public health implications.

## Background

Cinnamon is a common spice used by different cultures around the world for several centuries. It is obtained from the inner bark of trees from the genus Cinnamomum, a tropical evergreen plant that has two main varieties; *Cinnamomum zeylanicum* (CZ) and Cinnamon cassia (CC) (also known as *Cinnamomum aromaticum/*Chinese cinnamon). In addition to its culinary uses, in native Ayurvedic medicine Cinnamon is considered a remedy for respiratory, digestive and gynaecological ailments. Almost every part of the cinnamon tree including the bark, leaves, flowers, fruits and roots, has some medicinal or culinary use. The volatile oils obtained from the bark, leaf, and root barks vary significantly in chemical composition, which suggests that they might vary in their pharmacological effects as well [1]. The different parts of the plant possess the same array of hydrocarbons in varying proportions, with primary constituents such as; cinnamaldehyde (bark), eugenol (leaf) and camphor (root) [2]. Thus cinnamon offers an array of different oils with diverse characteristics, each of which determines its’ value to the different industries. For example the root which has camphor as the main constitute, has minimal commercial value unlike the leaf and bark [3]. It is this chemical diversity that is likely to be the reason for the wide-variety of medicinal benefits observed with cinnamon.

CZ, also known as Ceylon cinnamon (the source of its Latin name, zeylanicum) or ‘true cinnamon’ is indigenous to Sri Lanka and southern parts of India [3]. Three of the main components of the essential oils obtained from the bark of CZ are trans-cinnamaldehyde, eugenol, and linalool, which represent 82.5% of the total composition [4]. Trans-cinnamaldehyde, accounts for approximately 49.9–62.8% of the total amount of bark oil [5,6]. Cinnamaldehyde and eugenol are also the major components of CZ extracts [7]. A brief comparison of the two main varieties of cinnamon (CZ and CC) is included as a Additional file 1.

One important difference between CC and CZ is their coumarin (1,2-benzopyrone) content [8]. The levels of coumarins in CC appear to be very high and pose health risks if consumed regularly in higher quantities. According to the German Federal Institute for Risk Assessment (BfR), 1 kg of CC (CC) powder contains approximately 2.1-4.4 g of coumarin, which means 1 teaspoon of CC powder would contain around 5.8-12.1 mg of coumarin [9]. This is above the Tolerable Daily Intake (TDI) for coumarin of 0.1mg/kg body weight/day recommended by the European Food Safety Authority (EFSA) [10]. The BfR in its report specifically states that CZ contains ‘hardly any’ coumarin [9]. Coumarins are secondary phyto-chemicals with strong anticoagulant, carcinogenic and hepato-toxic properties [10]. The underlying mechanisms for the coumarin-related toxic effects are yet to be completely elucidated [10]. Due to the high concentrations in CC (compared with other foods), despite the relatively low amounts of the consumption of spices, studies have shown than coumarin exposure from food consumption is mainly due to CC [10]. The EFSA advocates against the regular, long term use of CC as a supplement due to its coumarin content [11]. In addition, according to currently available evidence coumarin does not seem to play a direct role in the observed biological effects of CC. Hence, although CC has also shown many beneficial medicinal properties, its’ coumarin content is likely to be an obstacle against regular use as a pharmaceutical agent, unlike in the case of CZ.

*In-vitro* and *in-vivo* studies in animals and humans from different parts of the world have demonstrated numerous beneficial health effects of CZ, such as anti-inflammatory properties, anti-microbial activity, reducing cardiovascular disease, boosting cognitive function and reducing risk of colonic cancer [12]. This paper aims to systematically review the scientific literature and provide a comprehensive summary on the potential medicinal benefits of ‘True Cinnamon’ (*Cinnamomum zeylanicum*). We also aim to provide a scientific guide to researchers on the potential areas for future research based on the positive findings obtained thus far from studies conducted by various research teams from around the world.

## Methods

A systematic review of published studies reporting the medicinal effects of CZ was undertaken in accordance with the PRISMA (Preferred Reporting Items for Systematic reviews and Meta-Analyses) statement guidelines (Additional file 2) [13]. A comprehensive search of the literature was conducted in the following databases; PubMed® (U.S. National Library of Medicine, USA), Web of Science® [v.5.3] (Thomson Reuters, USA), SciVerse Scopus® (Elsevier Properties S.A, USA) for studies published before 31^st^ December 2012. We used the following medical subject headings and keywords: “*Cinnamomum zeylanicum*”, “Ceylon cinnamon”, “True cinnamon” and “Sri Lankan cinnamon”. Results were limited to studies in English, while conference proceedings and commentaries were excluded.

In the second stage the total hits obtained from searching the databases using the above search criteria was pooled together and duplicate articles were removed. The remaining articles were initially screened by reading the ‘title’ and thereafter the ‘abstracts’. Studies not satisfying the inclusion criteria were excluded at these stages. The remaining articles were screened in the final stage by reading the full-text and those not meeting inclusion criteria were excluded. To obtain additional data a manual search was performed using the reference lists of included articles. Wherever possible forward citations of the studies retrieved during the literature search was traced and screened for possible inclusion. This search process was conducted independently by two reviewers (PR and SP) and the final group of articles to be included in the review was determined after an iterative consensus process.

## Results

### Literature search

**Figure 1:**
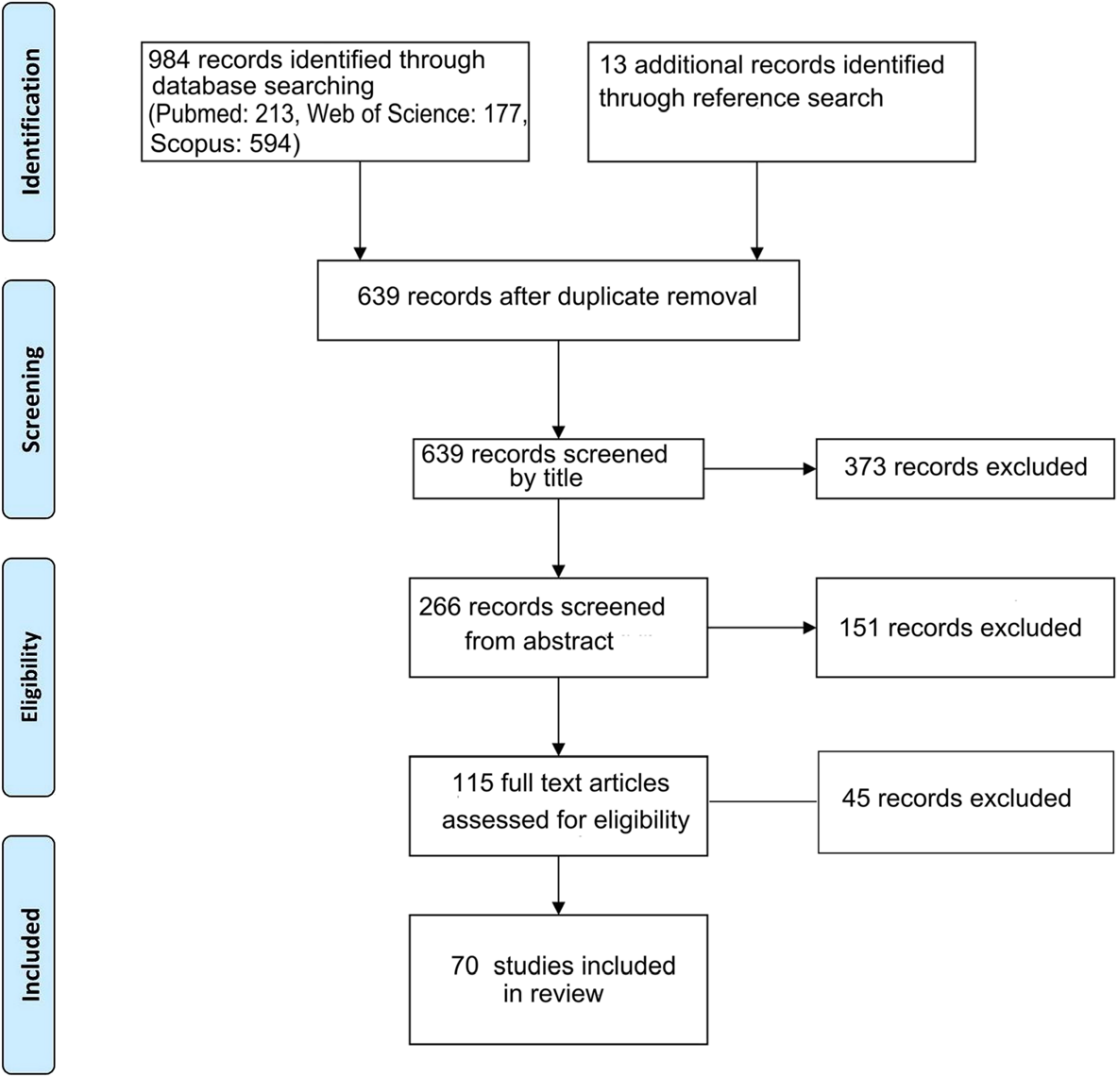
Summarized search strategy.

The literature search using the above search criteria identified the following number of articles in the respective databases; PubMed® (n = 54), Web of Science® (n = 76) and SciVerse Scopus® (n = 591). Thirteen additional articles were identified by manually searching the reference lists and forward citations of included papers. After removing duplicates the total number of articles included in the present review is 70. The search strategy is summarized in Figure 1.

### In-vitro and in-vivo anti-microbial properties

There were 30 different studies evaluating the *in-vitro* anti-microbial properties of CZ. Table 1 summarizes the findings of these studies. Accordingly CZ has shown potential anti-microbial action against a wide variety of bacteria *(Acinetobacter baumannii, Acinetobacter lwoffii, Bacillus cereus, Bacillus coaguiaris, Bacillus subtilis, Brucella melitensis, Clostridium difficile, Clostridium perfringens, Enterobacter aerogenes, Enterobacter cloacae, Enterococcus faecalis, Enterococcus faecium, Escherichia coli, Haemophilus Influenza, Helicobacter pylori, Klebsiella pneumonia, Listeria ivanovii, Listeria monocytogenes, Mycobacterium smegmatis, Mycobacterium tuberculosis, Proteus mirabilis, Pseudomonas aeruginosa, Saccharomyces cerevisiae, Salmonella typhi, Salmonella typhimurium, Staphylococcus albus, Staphylococcus aureus, Streptococcus agalactiae, Streptococcus pneumoniae, Streptococcus pyogenes and Yersinia enterocolitica).* In addition there seems to be activity against numerous fungi (*Aspergillus fiavus, Aspergillus fumigatus.*

**Table 1:** **Anti-microbial properties of *Cinnamomum zeylanicum***

**Author [ref]**	**Organism(s) tested**	**Main outcomes**
**Country(s)**		
Agasthya AS, et al. [14]	*Escherichia coli, Salmonella typhi, Salmonella paratyphi A/B, Brucella abortus and Brucella melitensis*	CZ extract were active only against *Brucella melitensis*
India		
Barattha MT, et al. [15]	*Clostridium perfringens, E. coli, Klebsiella pneumonia, Pseudomonas aeruginosa, S. aureus, Streptococcus faecalis and Yersinia enterocolitica*	Volatile oils from CZ had significant activity against the growth of food poisoning organisms, food spoilage organisms and organisms of faecal origin
UK, Italy, Portugal		
Bayoub K, et al. [16]	*Listeria monocytogenes, S. aurus, E. coli, Enterococcus faecalis, Klebsiella pneumoniae, Enterobacter cloacae, Acinetobacter baumannii*	CZ extracts demonstrated significant inhibitory effects on *S. aureus*, *Enterobacter cloacae, Acinetobacter baumannii and Listeria monocytogenes* (MIC 0.4 mg/ml)
Morocco		
Bhatia M, et al. [17]	*Candida albicans*	Among all spices tested CZ inhibited *C. albicans* most effectively (MIC 7.81 μl/ml)
India		
Carmo ES, et al. [18]	Aspergillus species (*A. fumigatus, A. niger, A. flavus, A. parasiticus, A. terreus and A. ochraceus*)	CZ essential oil possesses strong anti-aspergillus activity inhibiting the growth, spore germination and causing deleterious cellular morphological changes
Brazil		
Dubey RC, et al. [19]	*Salmonella typhi, Staphylococcus aureus, Escherichia coli, Klebsiella pneumoniae and Bacillus subtilis*	CZ essential oils inhibited growth of all organisms. Gram-negative organisms were more susceptible than gram-positive ones.
India		
Elumalai S, et al. [20]	*Bacillus subtilis, Klebsiella pneumonia, Pseudomonas aeruginosa, Staphylococcus aureus and Escherichia coli*	Percentage inhibition with CZ was; *B. Subtilis* (40.0%)*, Klebsiella pneumonia* (42.2%)*, Pseudomonas aeruginosa* (45.0%)*, Staphylococcus aureus* (37.8%) *and Escherichia coli* (40.0%). Inhibition activity of *C. Cassia* greater than CZ.
India		
Fabio A, et al. [21]	*S. pyogenes, S. agalactiae, S. pneumonia, Klebsiella pneumoniae, H. Influenza and S. aureus*	Of the 13 essential oils evaluated CZ and thyme showed the highest activity inhibiting all the strains studied
Italy		
Ferhout H, et al. [22]	*Malassezia furfur and Candida albicans*	Of the 3 oils studied CZ oil exhibited the strongest activity towards the two yeasts. M. furfur showed a greater sensitivity to CZ
France		
Gonçalves JLS, et al. [23]	Human rota-virus	CZ leaves and bark was able to inhibit the propagation of human rotavirus 32.4% and 33.9% respectively.
Brazil		
Guerra FQS, et al. [24]	Acinetobacter spp.	CZ essential oils suppresses the growth of Acinetobacter spp. and a synergistic effect was observed when combined with antibiotics
Brazil		
Hosseininejad Z, et al. [25]	*Helicobacter pylori*	CZ exhibited the most inhibitory effect on *H. pylori* and essential oils of CZ with IC_50_=0.3 μl/ml completely inhibited the growth of *H. pylori*.
Iran		
Jantan IB, et al. [26]	*Trichophyton mentagrophytes, T. tonsurans, T. rubrum, Microsporum canis, M. gypseum, M. audouini, Aspergillus fumigates, Candida albicans, C. glabrata, C. parapsilosis, C. tropicalis and Crytococcus neoformans*	Among all the essential oils, the leaf and bark oils of CZ showed the highest activity against all the fungi with MIC values of 0.04 to 0.63 μg μL−1
Malaysia		
Jirovetz L, et al. [27]	*Pseudomonas fluorescens*, *Escherichia coli* and S*taphylococcus aureus*	CZ essential oils were active against *E. coli and S. aureus*. However *P. fluorescens* was resistant.
Austria, Cameroon		
Khan R, et al. [28]	Multi drug resistant (MDR) strains of *Escherichia coli, Klebsiella pneumoniae and Candida albicans.*	The MDR strains were sensitive to the antimicrobial activity of CZ.
India		
Lima EO, et al. [29]	*Trichophyton rubrum, T. mentagraphytes, Microsporum canis and Epidermophyton floccosum*	CZ inhibited 80% of the dermatophyte strains tested and produced inhibition zones more than 10 mm in diameter
Brazil		
Maidment C, et al. [30]	*Escherichia coli B, staphylococcus albus and Saccharomyces cerevisiae*	CZ demonstrated microbial inhibitory effect; alcoholic extracts had greater activity than aqueous extracts. Essential oils had greater activity than the spices. MICs were smaller with the oils than with the spices.
UK		
Mandal S, et al. [31]	Methicillin resistant *Staphylococcus aureus*	CZ and *C. aromaticum* showed the strongest *in vitro* antibacterial activity against Methicillin Resistant *S. aureus*
India		
Mastura M, et al. [32]	*Trichophyton mentagrophytes, T. rubrum, Microsporum canis, Candida albicans and C. glabrata*	CZ was a moderate inhibitor of all the fungi tested (MIC values 1.26 – 2.51 μg/μl)
Malaysia		
Meades J, Jr et al. [33]	*Escherichia coli* (acetyl-CoA carboxylase inhibition)	CZ inhibited the carboxyl-transferase component of *E. coli* acetyl-CoA carboxylase enzyme.
USA, UK, South Africa		
Mishra AK, et al. [34]	*E. coli, Klebsiella pneumonia, Proteus vulgaris, Pseudomonas spp., S. aureus and S. pneumonia*	Of the 3 essential oils evaluated CZ oil showed the strongest inhibitory activity against all micro-organisms tested.
India		
Negi PS, et al. [35]	*Bacillus cereus, B. coaguiaris, B. subtilis, S. aureus, E. coli and Pseudomonas aeruginosa*	All crude extracts of CZ fruits showed antibacterial activity. Ethyl acetate and benzene extracts showed higher activity than methanol and water extract.
India, USA		
Noudeh GD, et al. [36]	*S. aureus, Bacillus subtilis, E. coli and Pseudomonas aeruginosa*	CZ inhibited the growth of all tested Gram- positive and Gram-negative strains.
Iran, UK		
Rana IS, et al. [37]	*Pseudomonas aeruginosa, S. aureus, Salmonella typhimurium and Bacillus subtilis*	Of the 19 essential oils evaluated the highest antibacterial activity was depicted by CZ against all bacteria
India		
Senhaji O, et al. [38]	*E. coli* O157:H7	In the presence of 0.05% of the oil, most of cells were killed after 30 min, suggesting a bactericidal action against *E. coli*. The MIC was around 625 ppm.
Morocco		
Shahverdi AR, et al. [39]	*Clostridium difficile*	The essential oil of CZ bark enhanced the bactericidal activity of clindamycin and decreased the MIC of clindamycin for *C. difficile.*
Iran		
Singh HB, et al. [40]	*Aspergillus niger. A. fumigatus. A. nididans, A. fiavus, Candida albicans, C, tropicalis, C, pseudotropicalis and Hisioplasma capsulatum*	Vapours of CZ bark oil and cinnamic aldehyde are effectively toxic at very low doses and at high inoculum density against the test fungi causing respiratory tract mycoses
India		
Sivakumar A, et al. [41]	*Mycobacterium tuberculosis*	Water (MIC-100 μg/ml) and ethanolic (MIC-200 μg/ml) extracts of CZ was observed to have activity against *M. tuberculosis*.
India		
Tekwu E, et al. [42]	*Mycobacterium tuberculosis* strains H37Rv and H37Ra	The MIC for H37Ra and H37Rv strains were 1024μg/ml and 512μg/ml respectively and MBC was >2048 μg/ml for both strains.
Cameroon, Turkey		
Unlu, M et al. [43]	*S. aureus, Streptococcus pyogenes, S. pneumonia, Enterococcus faecalis, Enterococcus faecium, Bacillus cereus, Acinetobacter lwoffii, Enterobacter aerogenes, E. coli, Klebsiella pneumoniae, Proteus mirabilis, Pseudomonas aeruginosa, Salmonella typhimurium, Clostridium perfringens, Listeria monocytogenes, Listeria ivanovii, Mycobacterium smegmatis, Candida albicans, Candida parapsilosis and Candida krusei*	The essential oil of CZ showed strong antimicrobial activity against all microorganisms tested,
Turkey		

*Aspergillus nididans, Aspergillus niger, Aspergillus ochraceus, Aspergillus parasiticus, Aspergillus terreus, Candida albicans, Candida glabrata, Candida krusei, Candida parapsilosis, Candida tropicalis, Crytococcus neoformans, Epidermophyton floccosum, Hisioplasma capsulatum, Malassezia furfur, Microsporum audouini, Microsporum canis Microsporum gypseum, Trichophyton mentagraphytes, Trichophyton rubrum and Trichophyton tonsurans).* CZ has also demonstrated activity against the human rota-virus (Table 1).

There were 5 studies evaluating *in-vivo* anti-microbial properties in animals. Abu, et al. [44] investigated the effect of administration CZ oil on the development and progression of the experimental cryptosporidiosis in mice, and they showed that administration of CZ oil was beneficial in protecting susceptible hosts against opportunistic zoonotic parasites such as *Cryptosporidium parvum*. Rosti, et al. [45,46] reported two cases of infants who were chronic carriers of *Salmonella enteritidis* who received short term (10 days) administration of grounded CZ bark which led to consistently negative stool cultures and no clinical or microbiological relapses. Activity of CZ against fluconazole resistant and susceptible candida were studied in HIV infected patients having pseudo-membranous Candida, where 3 patients out of 5 showed improvements in their oral candidiasis [47]. The effects of sugared chewing gum containing cinnamic aldehyde and natural flavours from CZ on the short-term germ-killing effect on total and H_2_S-producing salivary anaerobes was investigated by Zhu, et al. [48]. Significant reductions in total salivary anaerobes and H_2_S-producing salivary anaerobes were observed 20 minutes after subjects chewed the gum.

### In-vitro and in-vivo anti-parasitic effects

Samarasekera, et al. [49] investigated the mosquito control properties of essential oils of leaf and bark of CZ against Culex *quinquefasciatus*, Anopheles *tessellatus* and *Aedes aegypti*. CZ bark oil showed good knock-down and mortality against *A. tessellatus* (LD_50_ 0.33 μg/mL) and *C. quinquefasciatus* (LD_50_ 0.66 μg/mL) than leaf oil (LD_50_ 1.03 and 2.1 μg/mL). Yang, et al. [50] showed that CZ bark essential oil was slightly less effective than either d-phenothrin or pyrethrum against eggs and adult females of human head louse, *Pediculus humanus capitis*, using direct contact and vapour phase toxicity bioassays.

### In-vitro and in-vivo effects on blood pressure, glycaemic control and lipids

A recent meta-analysis by Ranasinghe, et al. and a systematic review by Bandara et al., on the effects of CZ extracts on diabetes demonstrates numerous beneficial effects both *in-vitro* and *in-vivo*[51,52]. *In-vitro* CZ has demonstrated a potential for; a) reducing post-prandial intestinal glucose absorption by inhibiting the activity of enzymes involved in carbohydrate metabolism (pancreatic α–amylase and α–glucosidase), b) stimulating cellular glucose uptake by membrane translocation of GLUT-4, c) stimulating glucose metabolism and glycogen synthesis, d) inhibiting gluconeogenesis by effects on key regulatory enzymes and f) stimulating insulin release and potentiating insulin receptor activity [51]. Cinnamtannin B1 was identified as the potential active compound responsible for these effects [51]. The beneficial effects of CZ *In-vivo* includes; a) attenuation of weight loss associated with diabetes, b) reduction of Fasting Blood Glucose, c) reducing LDL and increasing HDL cholesterol, d) reducing HbA1c and e) increasing circulating insulin levels [51]. In addition CZ also showed beneficial effects against diabetic neuropathy and nephropathy [51].

Hasan et al. [53], also confirmed these effects and demonstrated that CZ reduced total cholesterol, LDL cholesterol and triglycerides while increasing HDL-cholesterol in diabetic rats. Similar results have also been observed in hyper-lipidaemic albino rabbits [54]. However, feeding CZ to animals at levels corresponding to the average human dietary intake has not shown to reduce lipid levels significantly [55]. Nyadjeu et al. [56] examined the effects of CZ extracts (CZA) on mean arterial blood pressure (BP) of normotensive (NR) rats, salt-loaded hypertensive rats (SLHR), L-NAME hypertensive rats (LNHR) and spontaneously hypertensive rats (SHR). Immediately after intravenous administration a significant drop of BP was shown in NTR, SLHR and LNHR in a dose dependent manner, the drop in BP was not dose dependent in SHR [56]. Wansi, et al. demonstrated similar effects in NTR and SLHR, they also showed that CZ has a vaso-relaxant effect on the rat thoracic aortic ring segments, suggesting that, CZ might be inhibiting extracellular Ca^2+^ through L-type voltage-sensitive channels [57]. Markey, et al. [58] tested the hypothesis that supplementing a single high fructose breakfast with 3g of cinnamon would delay gastric emptying of a high-fat solid meal utilizing the ^13^C octanoic acid breath test, and consequently reduce postprandial blood glucose and lipid concentrations. There concluded that cinnamon did not change gastric emptying parameters, postprandial triacylglycerol or glucose concentrations after a single administration [58]. It is important to note that all *in-vivo* studies except the above study were conducted in animals.

### In-vitro and in-vivo anti-oxidant properties

The essential oils obtained from the bark of CZ and eugenol has shown very powerful activities, decreasing 3-nitrotyrosine formation and inhibiting the peroxynitrite-induced lipid peroxidation in *in-vitro* assays [59]. The volatile oils of CZ has shown 55.9% and 66.9% antioxidant activity at 100 and 200 ppm concentration, respectively [60]. The dried fruit extracts of CZ with ethyl acetate, acetone, methanol and water exhibited antioxidant activity in the order of water > methanol > acetone > ethyl acetate [61]. The etheric (0.69 mg), methanolic (0.88 mg) and aqueous (0.44 mg) cinnamon extracts, inhibited the oxidative process in 68%, 95.5% and 87.5% respectively [62]. A. Kitazuru, et al. [63] studied the effects of ionizing radiation on natural CZ antioxidants and showed that irradiation in the dose range applied did not have any effect on the antioxidant potential of the cinnamon compounds.

CZ bark extracts were found to be potent in free radical scavenging activity especially against DPPH radicals and ABTS radical cations, while the hydroxyl and superoxide radicals were also scavenged by the tested compounds [64]. Similar findings were noted by Prakash, et al. who showed that CZ has 65.3% of anti-oxidant activity and strong free radical scavenging activity [65]. Ranjbar, et al. [66] treated 18 operating room personnel with CZ (100 mg/300 mL tea) daily for 10 days and blood samples were analyzed for biomarkers of oxidative stress biomarkers including Lipid Peroxidation Level (LPO), Total Antioxidant Power (TAP) and Total Thiol Molecules (TTM). Treatment of subjects with cinnamon induced a significant reduction in plasma LPO, however no statistically significant alteration was found for plasma TAP and TTM after 10 days treatment with CZ [66]. Treatment of 54 healthy volunteers with CZ 100 mg/30ml of tea daily were significantly effective in the reduction of lipid peroxidation and increasing TAP and TTM in comparison with controls [67]. The extent of increase in plasma TBARS and TAP for the CZ group was significantly higher than in those give regular tea only [67].

### Other in-vitro effects

An aqueous extract of CZ is known to inhibit tau aggregation and filament formation, which are hallmarks of Alzheimer’s disease [68]. The extract also promotes complete disassembly of recombinant tau filaments and cause substantial alteration of the morphology of paired-helical filaments isolated from brains of those with Alzheimer’s disease, however it was not deleterious to the normal cellular function of tau. An A-linked proanthocyanidin trimer molecule isolated from the CZ extract has shown to contain a significant proportion of this inhibitory activity [68]. Takasao, et al. [69] demonstrated that CZ extracts facilitates collagen biosynthesis in human dermal fibroblasts. CZ extract up-regulated both mRNA and protein expression levels of type I collagen without cytotoxicity, cinnamaldehyde was the major active component promoting the expression of collagen by HPLC and NMR analysis. This suggests that CZ extracts might be useful in anti-aging treatment of skin [69]. CZ extracts have also exhibited the strong inhibitory effects on osteoclastogenesis [70]. CZ dose-dependently inhibited osteoclast-like cell formation at concentrations of 12.5-50 μg/ml without affecting cell viability. This finding raises prospects for the development of a novel approach in the treatment of osteopenic diseases [70].

### Other in-vivo effects in animals

CZ is known to have anti-secretagogue and anti-gastric ulcer effects as shown by a study conducted by Alqasoumi [71]. CZ suspension pre-treatment decreased the basal gastric acid secretion volume in pylorus ligated rats and it effectively inhibited gastric hemorrhagic lesions induced by 80% ethanol, 0.2M NaOH, and 25% NaCl. It also showed antiulcer activity against indomethacin. CZ treatment replenished the ethanol-induced decreased levels of gastric wall mucus [71]. Rao and Lakshmi induced diarrhoea in mice using the magnesium sulphate-induced diarrhoea test and showed that CZ extracts at 100 and 200 mg/kg doses significantly reduced the extent of the diarrhoea (71.7% and 80.4%) in test animals [72].

In a study using two animal models for the investigation of the anti-nociceptive and anti-inflammatory effects of CZ and selected plants, CZ induced a dose-dependent analgesic protective effect against both thermal stimuli and the writhing syndrome, furthermore, CZ showed an anti-inflammatory effect against chronic inflammation induced by cotton pellet granuloma indicating anti-proliferative effect [73]. These effects have been verified by other authors [74]. CZ is also known to have wound healing properties in rats, in a study using thirty-two rats where experimental excision wounds were induced and treated with topical CZ containing ointments. The CZ extracts served to accelerate the wound healing process and specifically increased epithelialisation [75]. In Wister rats CZ given orally increased the wound breaking strength significantly in incision wounds model and in dead space wounds the granulation tissue breaking strength and hydroxyproline content were significantly increased [76].

CZ has also been shown to have hepato-protective effects in a study where liver injury was induced in rats by CCl_4_[77]. Administration of CZ extracts (0.01, 0.05 and 0.1 g/kg) for 28 days significantly reduced the impact of CCl_4_ toxicity on the serum markers of liver damage (AST, ALT and ALP). In addition, treatment with CZ markedly increased the levels of superoxide dismutase and catalase enzymes in rats [77]. Water-based extract from CZ was a potent inhibitor of VEGFR2 kinase (Vascular Endothelial Growth Factor Receptor) activity which is involved in angiogenesis [78]. As a result, CZ inhibited VEGF-induced endothelial cell proliferation, migration and tube formation *in-vitro*, sprout formation from aortic ring *ex-vivo* and tumor-induced blood vessel formation *in-vivo*[78].

### Toxic effects

*In-vivo* studies in animals have also highlighted lack of significant toxic effects on liver and kidney, with a significantly high therapeutic window [51]. Domaracký et al., [79] administered CZ for two weeks to female mice and evaluated the effects on the viability of embryos of mice, number of nuclei and the distribution of embryos according to nucleus number. Cinnamon significantly decreased the number of nuclei and the distribution of embryos according to nucleus number was significantly altered and these changes were attributed to the anti-proliferative effects of cinnamaldehyde [79]. However these findings have been contradicted by others who have demonstrated that CZ does not have significant abortive or embryo toxic effects in animals [80]. Furthermore, Shah et al, showed that CZ induced a significant increase in reproductive organ weights, sperm motility, sperm count and demonstrated no spermatotoxic effects in mice [81].

Chulasiri et al., demonstrated that petroleum ether and chloroform extracts from CZ showed cytotoxic effects on KB (human mouth carcinoma cell line) and L1210 cells (mouse lymphoid leukaemia cell line) [82]. The average ED_50_ from the first and second tests of the petroleum ether extract on these tumour cells were 60 and 24 pg/ml respectively and of the chloroform extract were 58 and 20 pg/ml respectively. Singh, et al. [83] investigated the cytotoxic effects of aqueous cinnamon extract from the bark of CZ on human and mouse cell lines. The aqueous cinnamon extract proved to be more cytotoxic to cancerous cells at concentrations just above 0.16 mg/mL. At a critical concentration of 1.28 mg/mL, CZ treatment resulted in 35-85% growth inhibition of the majority of the cancerous cells.

## Discussion

The available *in-vitro* and *in-vivo* evidence suggests that CZ has anti-microbial, anti-parasitic, anti-oxidant and free radical scavenging properties. In addition CZ seems to lower blood glucose, serum cholesterol and blood pressure, suggesting beneficial cardiovascular effects.

The different parts of the CZ plant possess the same array of hydrocarbons in varying proportions. This chemical diversity is likely to be the reason for the wide-variety of medicinal benefits observed. It would also be interesting to identify probable mechanisms that are responsible for such a wide array of medicinal benefits. The mechanism of action by which CZ reduces blood glucose has been well studied *in-vitro* and *in-vivo*, it seems that CZ; a) reduces intestinal glucose absorption by inhibiting enzymes, b) stimulates cellular glucose uptake, glycogen synthesis, insulin release and potentiates insulin receptor activity and c) inhibits gluconeogenesis by effects on key regulatory enzymes.

The mechanism for the lipid lowering effects is not clearly described in literature. The high dietary fibre content of CZ could result in reduced intestinal lipid absorption, and the high vitamin/anti-oxidant is likely to result in increased lipid metabolism. Insulin plays a key role in lipid metabolism and it is possible that increased serum Insulin levels following CZ administration also contributes towards reducing lipid levels. The exact blood pressure-lowering mechanism of cinnamon is still unknown and new studies are needed to clarify this issue. The results of studies in animals have indicated that cinnamon regulates blood pressure levels through peripheral vasodilatation [84]. This vasodilatation might be partially through Ca^2+^ channels blocking properties [57].

The phenolic constituents of CZ are likely to be responsible for the anti-oxidant and free radical scavenging activity observed. Cinnamon extracts are known to increase Tristetraprolin mRNA and protein levels, Tristetraprolins have anti-inflammatory effects due to destabilizing of pro-inflammatory mRNA [85]. This could be the reason for the anti-inflammatory actions observed. The anti-microbial action is considered to arise mainly from the potential of hydrophobic essential oils to disrupt the bacterial cell membrane and its structures which leads to ion leakage [37]. Antibacterial assays of the column chromatography fractions clearly indicated that cinnamaldehyde is the primary compound responsible for major antibacterial activity [37]. Trans-cinnamaldehyde is also known to inhibits bacterial acetyl-CoA carboxylase [33].

We acknowledge several limitation to the extent to which conclusions can be drawn from the present systematic review. The CZ specimen was either not authenticated or authentication details were not mentioned in majority of the studies, however considering that a majority of the studies were conducted in countries where CZ is cultivated, it is likely that the species used were ‘True’ cinnamon. There were minimal studies evaluating the effects of CZ in humans and majority of the studies were *in-vitro* or *in-vivo* in animals, hence care needs to be drawn when generalizing the conclusions to the human population. In order to have public health implications these effects need to be reproducible in humans. Lack of well-designed human trials has compromised our knowledge on common side-effects, drug interactions and efficacy in humans. Further randomized double-blinded placebo-controlled clinical trials are required to establish therapeutic safety and efficacy of CZ as a pharmaceutical agent.

## Conclusions

The available *in-vitro* and *in-vivo* evidence suggests that CZ has anti-microbial, anti-parasitic, anti-oxidant and free radical scavenging properties. In addition CZ seems to lower blood glucose, serum cholesterol and blood pressure, suggesting beneficial cardiovascular effects. However, randomized controlled human trials will be necessary to determine whether these effects have public health implications.

## Competing interest

The authors declare that they have no competing interests.

## Authors’ contributions

PR, GASP, PG, GRC and PK made substantial contribution to conception and study design. PR and SP were involved in data collection. PR, SP, GASP and GRC were involved in refining the study design, statistical analysis and drafting the manuscript. PR, PG and PK critically revised the manuscript. All authors read and approved the final manuscript.

## Pre-publication history

The pre-publication history for this paper can be accessed here:

http://www.biomedcentral.com/1472-6882/13/275/prepub

## Supplementary Material

Additional file 1 A brief comparison of the two main varieties of cinnamon (Cinnamomum zeylanicum and Cinnamomum cassia). Click here for file

Additional file 2 PRISMA (Preferred Reporting Items for Systematic reviews and Meta-Analyses) Checklist. Click here for file
