# Supplementary material for: Medicinal properties of ‘true’ cinnamon (Cinnamomum zeylanicum): a systematic review
Source: BMC Complement Altern Med. 2013 Oct 22;13:275. doi: 10.1186/1472-6882-13-275 (PMC3854496; doi:10.1186/1472-6882-13-275)
Supplement: Additional file 2 — PRISMA (Preferred Reporting Items for Systematic reviews and Meta-Analyses) Checklist. [file 1472-6882-13-275-S2.doc]

|  | *Cinnamomum zeylanicum* (CZ) | *Cinnamomum cassia* (CC) |
| --- | --- | --- |
| Other common names | *Cinnamomum verum*, Ceylon cinnamon, Sri Lanka cinnamon and true cinnamon | *Cinnamomum aromaticaum,* Chinese cinnamon, Chinese cassia, and Saigon cinnamon |
| Areas of cultivation | Sri Lanka (80-90% of world’s supply), Madagascar, Southern India and Seychelles | China, India, Indonesia, Laos, Malaysia, Taiwan, Thailand and Vietnam |
| Main Constituents  Stem bark oils    Root bark oils    Leaf oils    Fruit oils | trans-cinnamaldehyde (49.9–62.8%), eugenol, cinnamyl acetate, linalool and benzyl benzoate  camphor (up to 60%), 1,8-Cineole, eugenol, terpinol  cinnamaldehyde  eugenol (60-90%), cinnamaldehyde, linalool and cinnamyl acetate  cadinene (30-40%), cadinol and ß-caryophyllene, | CC usually produces only one main type of oil, almost 95% of this oil consists of cinnamaldehyde with slight variation between the different parts of the plant. Other constituents include methoxycinnamaldehyde, benzaldehyde, coumarin, limonene, eugenol and cinnamyl acetate  (Methods of distinguishing CZ oil from CC oil is based on the presence of increased content of benzaldehyde, methoxycinnamaldehyde and coumarin in CC oil) |
| Medicinal properties | - Anti-microbial properties - Anti-parasitic properties - Anti-oxidant & free radical scavenging properties - Anti-diabetic properties - Reduction in serum cholesterol - Reduction in blood pressure | - Anti-microbial properties - Antioxidant properties - Anti-diabetic properties - Anti-cancer properties |
| Toxicology | CZ contained only traces (0.004%) or no detectable levels of coumarin | Generally CC contains up to 1% coumarin. Coumarins are plant compounds with strong anticoagulant, carcinogenic and hepato-toxic properties. Coumarin is known to cause liver and kidney damage in rats and mice and there are isolated incidents of similar hepato-toxicity in humans |
